# Impact of SARS-CoV-2 Infection on Essential Tremor: A Retrospective Clinical and Kinematic Analysis

**DOI:** 10.1007/s12311-024-01751-5

**Published:** 2024-10-09

**Authors:** Davide Costa, Sofia Grandolfo, Daniele Birreci, Luca Angelini, Massimiliano Passaretti, Antonio Cannavacciuolo, Adriana Martini, Martina De Riggi, Giulia Paparella, Alfonso Fasano, Matteo Bologna

**Affiliations:** 1 IRCCS Neuromed, Pozzilli (IS), Italy; 2 Department of Human Neurosciences, Sapienza University of Rome, Rome, Italy; 3 Krembil Research Institute, University Health Network, Toronto, ON Canada; 4 Edmond J. Safra Program in Parkinson’s Disease, Morton and Gloria Shulman Movement Disorders Clinic, Toronto Western Hospital, Division of Neurology, University of Toronto, Toronto, ON Canada; 5 Department of Clinical Neuroscience, Karolinska Institutet, Solna, Sweden

**Keywords:** Essential tremor, SARS-CoV-2, Worsening

## Abstract

In the past few years, SARS-CoV-2 infection has substantially impacted public health. Alongside respiratory symptoms, some individuals have reported new neurological manifestations or a worsening of pre-existing neurological conditions. We previously documented two cases of essential tremor (ET) who experienced a deterioration in tremor following SARS-CoV-2 infection. However, the effects of SARS-CoV-2 on ET remain largely unexplored. This study aims to evaluate the impact of SARS-CoV-2 infection on a relatively broad sample of ET patients by retrospectively comparing their clinical and kinematic data collected before and after the exposure to SARS-CoV-2. We surveyed to evaluate the impact of SARS-CoV-2 infection on tremor features in ET. Subsequently, we retrospectively analysed clinical and kinematic data, including accelerometric recordings of postural and kinetic tremor. We included 36 ET patients (14 females with a mean age of 71.1 ± 10.6 years). Among the 25 patients who reported SARS-CoV-2 infection, 11 (44%) noted a subjective worsening of tremor. All patients reporting subjective tremor worsening also exhibited symptoms of long COVID, whereas the prevalence of these symptoms was lower (50%) in those without subjective exacerbation. The retrospective analysis of clinical data revealed a tremor deterioration in infected patients, which was not observed in non-infected patients. Finally, kinematic analysis revealed substantial stability of tremor features in both groups. The study highlighted a potential correlation between the SARS-CoV-2 infection and clinical worsening of ET. Long COVID contributes to a greater impact of tremor on the daily life of ET patients.

## Introduction

The severe acute respiratory syndrome coronavirus 2 (SARS-CoV-2) infection has had a profound impact on people’s lives over the past few years. In addition to the typical clinical manifestations of coronavirus disease 2019 (COVID-19), neurological symptoms have also been frequently reported [1–4]. Neurological manifestations were especially prominent in hospitalized patients with severe COVID-19 and those with pre-existing pathologies [5–8]. Again, the issue of long-term consequences in patients affected by COVID-19, commonly referred to as ‘long COVID’, has come to the forefront. Long COVID is defined as a multisystemic condition with signs and symptoms that develop after an infection consistent with COVID-19. Notably, long COVID also includes neurological manifestations, such as cognitive impairment, fatigue, muscle pain, or anxiety [9–11]. Finally, individuals with pre-existing neurological conditions may experience a worsening of their symptoms after SARS-CoV-2 infection. Numerous studies examining the impact of COVID-19 on chronic neurological conditions, including Parkinson’s disease (PD) and hyperkinetic disorders, have highlighted how the infection can lead to an exacerbation of symptoms in these conditions [12–14]. In this context, we have previously documented two cases of essential tremor (ET) worsening following SARS-CoV-2 infection [15–17]. While supported by a thorough clinical evaluation and an objective assessment of tremor before and after the infection, it is essential to note that these cases are anecdotal. Further investigation is needed to explore the potential effects of SARS-CoV-2 infection on ET more comprehensively.

In this study, we aimed to assess the impact of SARS-CoV-2 infection on a sample of patients diagnosed with ET. We retrospectively compared their clinical and kinematic data collected before and after exposure to SARS-CoV-2 and compared the results with those of ET patients who never experienced SARS-CoV-2 infection. Furthermore, we explored other neurological manifestations that may have emerged following the pandemic, including symptoms associated with long COVID. The findings from this study may carry clinical and pathophysiological implications.

## Methods

### Participants and Telephone Survey

Between May and July 2023, S.G. conducted a telephone survey on a cohort of patients diagnosed with ET based on the most recent criteria [18], recruited from the Movement Disorders outpatient clinic at the Department of Human Neurosciences, Sapienza, University of Rome. The surveyed patients had been previously involved in a longitudinal evaluation performed by G.P. and L.A., including periodic clinical and/or kinematic evaluations of tremor spanning from August 2018 to May 2023. The telephone survey, specially designed for this study, drew inspiration from previous research on the impact of COVID-19 [19] consisting of nineteen questions. The survey aimed to investigate the effects of SARS-CoV-2 infection on tremor features. In brief, patients were asked about their experiences with SARS-CoV-2 infection within the past three years, diagnosed through an antigenic nasopharyngeal swab. Questions covered the severity of infection-related symptoms, the need for oxygen therapy, hospitalization, and the duration of recovery. Patients who had experienced SARS-CoV-2 infection were further questioned about subjective changes in tremor features, including tremor amplitude, persistence throughout the day, or occurrence in previously unaffected body segments. Additionally, the survey explored other neurological symptoms, encompassing those associated with ‘long COVID’ [9–11], such as fatigue, myalgia, and ‘brain fog’, defined as difficulty thinking or concentrating. The experimental procedures adhered to the principles of the Declaration of Helsinki and were approved by the local ethics committee. All participants provided informed consent to participate in the study.

### Clinical and Kinematic Data Analysis

D.C. conducted a retrospective analysis of clinical and kinematic data for patients who, based on the telephone survey, had experienced SARS-CoV-2 infection between two consecutive tremor evaluations. These evaluations included a baseline assessment (before the infection, T0) and the first assessment conducted after the infection (T1). A retrospective analysis of clinical and kinematic data collected in two different experimental sessions (T0’ and T1’) was also conducted in ET patients who responded to the telephone survey by stating that they had never been affected by the SARS-CoV-2 infection. Both the clinical and kinematic had been carried out after the discontinuation for at least 12 h of tremor treatments to minimize the role of medication.

The clinical assessment used the Fahn-Tolosa-Marin Clinical Rating Scale for Tremor (FTMTRS) [20]. This scale is divided into three parts: in the first part (Section A), tremor amplitude is assessed in nine different body parts at rest, during posture holding, and while performing specific actions. The second part (Section B) evaluates action tremors during tasks such as writing, drawing a spiral, and tracing straight lines. The third part (Section C) focuses on functional disability. We also tested cognitive and psychiatric functions in patients using the Mini-Mental State Examination (MMSE), Hamilton Anxiety Rating Scale (HAM-A) and Hamilton Depression Rating Scale (HAM-D).

The kinematic assessments were conducted using the SMART motion system by BTS Engineering (Milan, Italy), consisting of three infrared cameras operating at 120 Hz. These cameras accurately capture motion in the 3D space by tracking reflective markers taped to different body segments. This methodology ensures a consistent approach to assessing upper limb tremor during postural and kinetic tasks, as employed in prior assessments [15, 16, 21–26]. We recorded upper limb postural tremor during two postures: (i) with the arms outstretched in front of the chest (posture 1, P1), and (ii) with the arms flexed at the elbows, commonly known as ‘wing beating’ posture (posture 2, P2). Additionally, upper limb kinetic tremor was recorded during a ‘pointing task’, during which participants repetitively move their index finger from their nose to a reflective target fixed on a support approximately 15 cm above the table at sternal height and positioned at about 2/3 arm distance. Three 15-second recordings were conducted for each task [15, 16, 21–26]. Tremor analysis was conducted using dedicated software (SMART Analyzer, BTS Engineering). For postural tremor of the upper limbs, the signal was filtered with a bandpass filter at 3–12 Hz. The power spectrum was then obtained for each track using a Welch periodogram with a segment length of two seconds and a Hammer taper. Tremor was considered present in a track if a clear peak with half-power bandwidth narrower than 2 Hz was present [27]. The tremor frequency peak expressed in Hz was selected for each patient, considering the average value of the three axes in the different tracks when differences existed. The tremor amplitude was then determined by measuring the tremor power at the individual frequency peak ± 1 Hz in the three axes of space, and calculating the magnitude of the accelerometer vector with the formula $\:\sqrt{{x}^{2}+{y}^{2}+{z}^{2\:}}$ [28]. The average amplitude values of tracks were considered and expressed in the squared acceleration root mean square (GRMS2). Regarding the kinetic tremor of the upper limb, we employed the curvature index (CI), defined as the arm endpoint average path length divided by the length of a straight line joining the initial and final positions [23, 25]. The average of the values from both body sides and postures P1-P2 was considered for postural and kinetic tremor assessments [15, 16, 21–26]. 

### Statistical Analysis

Categorical variables were expressed as frequencies and compared using the Fisher’s exact and the McNemar’s test, where appropriate. Numerical variables recorded at T0 and T1 in each of the two subgroups of patients (infected and non-infected) were compared using Wilcoxon signed-rank test, including a baseline comparison between infected and non-infected patients. Kinematic parameters recorded at T0/T0’ and T1/T1’ were compared using paired t-tests. We calculated the individual longitudinal variation coefficient using total FTMTRS scores in both infected and non-infected patients [Δ= (T1/T1’) - (T0/T0’)]. Subsequently, we identified patients within each subgroup whose FTMTRS total scores deteriorated by more than one standard deviation (SD) from the mean baseline score and compared the frequencies using the Fisher’s exact test. In infected patients, we calculated the time to infection (TTI) by dividing the elapsed time between the T0 assessment and the infection by the longitudinal T0-T1 observation time. Possible correlations between the longitudinal variation coefficient of clinical and kinematic values and the TTI were tested using the Spearman coefficient. All results are presented as mean value ± 1 SD unless otherwise specified. The level of significance was set at *p* < 0.05. Data were analysed using SPSS Statistics for Windows v26.0 (IBM, released 2019).

## Results

### Telephone Survey

**Fig. 1:**
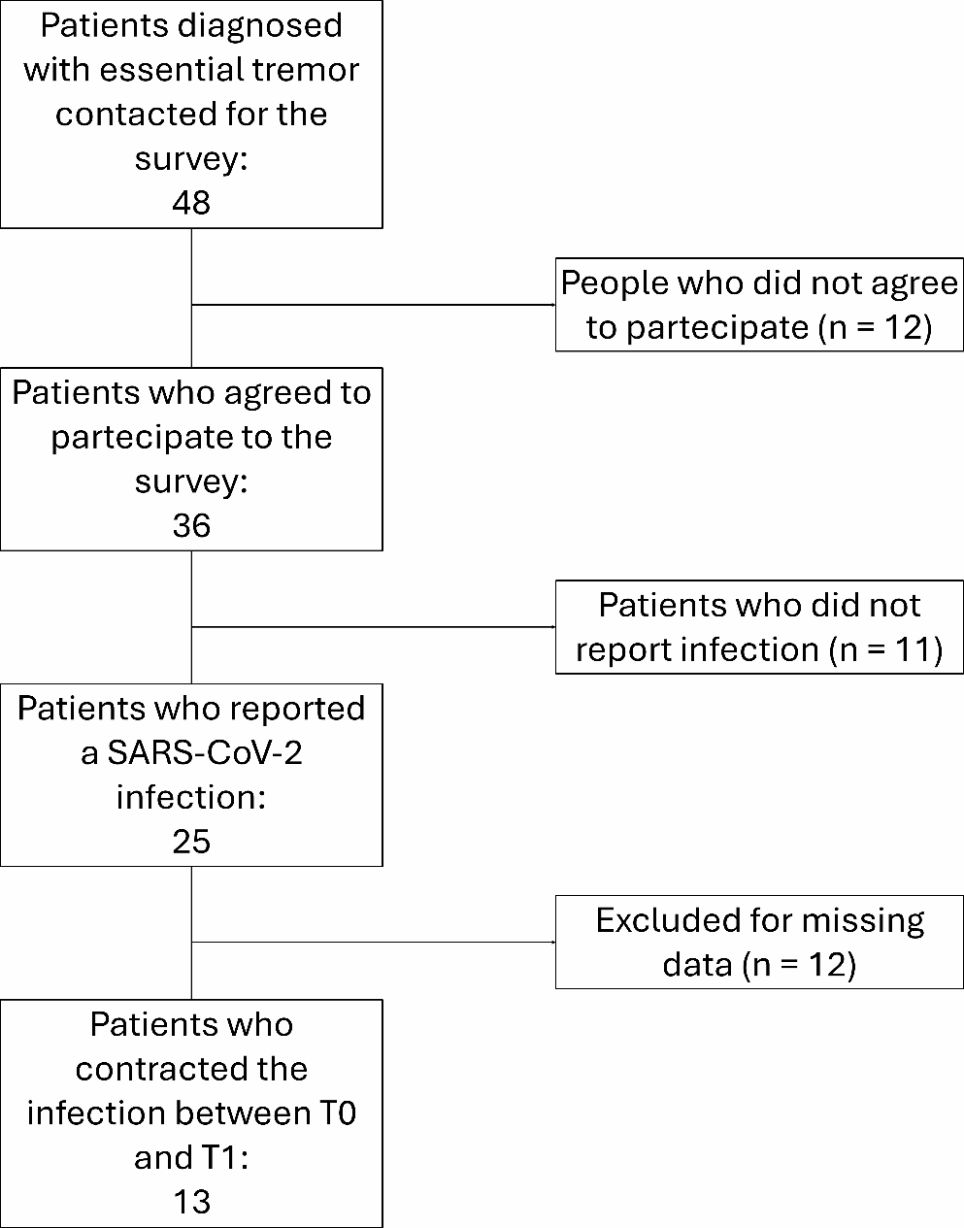
The flowchart shows the number of patients initially recruited, those excluded for various reasons and the final sample

We reached out to 48 patients, all of whom had already undergone clinical and kinematic evaluation during longitudinal assessments. Among these 48 patients, 36 (75%) agreed to participate in the study (Fig. 1). The study population consisted of 14 females and 22 males, averaging 71.1 ± 10.6 years. Within the 36 patients included in the study, 25 (69%) had reported a SARS-CoV-2 infection within the past three years, confirmed by antigenic nasopharyngeal swabs (Fig. 1). Notably, none of these patients had exhibited severe disease manifestations, and none had required hospitalization or oxygen therapy. In all cases, COVID-19-related respiratory symptoms had resolved within three weeks.

Among the 25 patients who reported a SARS-CoV-2 infection, 11 (44%) subjectively experienced tremor worsening (Fig. 1). Specifically, 10 patients (40%) reported increased tremor amplitude and 8 (32%) noted longer-lasting tremors throughout the day. One patient (4%) reported the emergence of tremor in a previously unaffected body part. The deterioration of tremor characteristics following SARS-CoV-2 infection in these patients appeared to persist at the time of the survey, with only 2 of the 11 patients reporting subsequent improvement in tremor that returned to its pre-infection baseline condition. Among the patients examined, 18 (72%) reported long COVID symptoms, such as cognitive disturbances, fatigue and myalgia. Notably, all patients (100%) who reported a subjective worsening tremor also experienced long COVID symptoms. In contrast, the prevalence of these symptoms was significantly lower (50%) in those without subjective tremor exacerbation.

### Retrospective Analysis of Clinical and Kinematic Data

Thirteen out of the 25 patients (52%) exposed to SARS-CoV-2 contracted the infection within the evaluation period, i.e., between T0 and T1 (Fig. 1). We excluded 12 ET patients who reported in the telephone survey a SARS-CoV-2 infection, however this did not coincide with the time frame between the two available tremor assessments.

At baseline, the analysis did not show any significant differences in terms of demographic and clinical features between infected and non-infected patients (age: 69.8 ± 12.3 vs 72.6 ± 8.6 years old, *p* = 0.73; MMSE: 27.4 ± 1.0 vs 27.7 ± 1.0, *p* = 0.39; HAM-A: 8.0 ± 6.8 vs 10.0 ± 6.9, *p* = 0.61 and HAM-D: 7.8 ± 6.9 vs 10.5 ± 6.2, *p* = 0.33). Additionally, the historical and clinical characteristics of tremor were similar between the two subgroups at T0/T0’ (family history of tremor: 76.9% vs 45.5%, *p* = 0.21 by Fisher’s exact test; age of onset: 48.2 ± 18.5 vs 54.4 ± 16.0 years old, *p* = 0.57; tremor duration: 16.0 ± 15.8 vs 12.2 ± 12.1 years, *p* = 0.91 and FTMTRS total scores: 18.9 ± 12.0 vs 22.2 ± 13.0, *p* = 0.49).

**Fig. 2:**
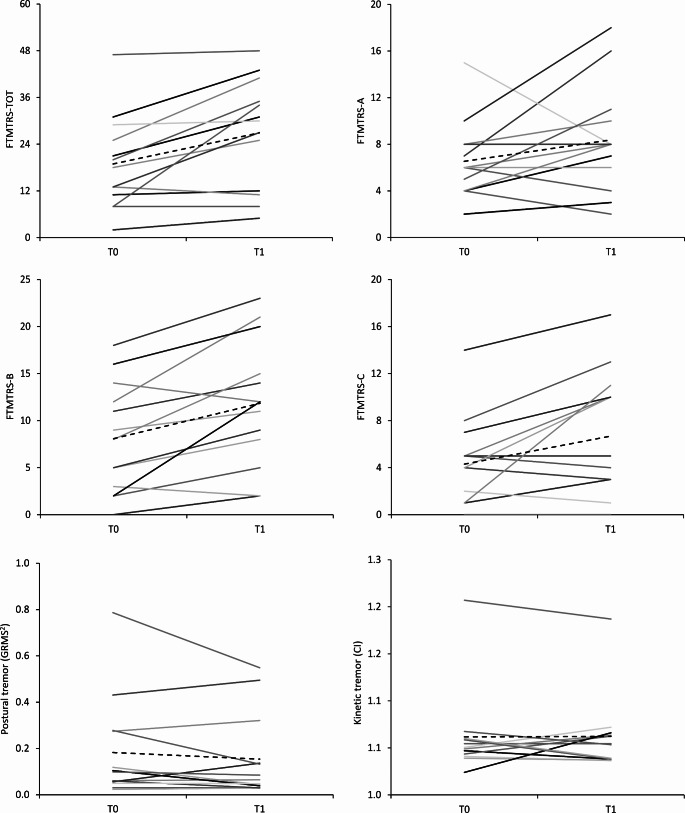
Upper and middle panels: Fahn-Tolosa-Marin Clinical Rating Scale for Tremor (FTMTRS) scores and subscores. Lower panel: postural (left) and kinetic (right) tremor amplitude as kinematically evaluated. Data refers to infected patients. Solid lines correspond to individual data and dashed lines to average values

**Fig. 3:**
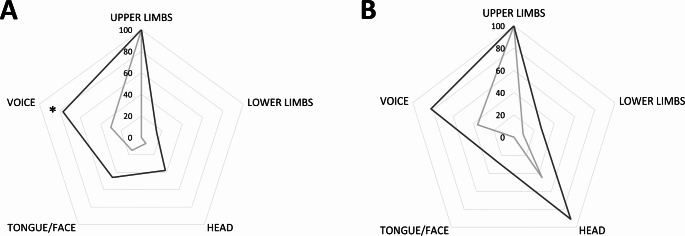
Percentage of tremor distribution in different body segments in ET patients with (**A**) and without (**B**) reported SARS-CoV-2 infection at T0/T0’ (grey line) and at T1/T1’ (black line) evaluations are shown

The average time between T0 and T1 in infected patients was 38.5 ± 16.3 months; the average time between T0 and the infection was 31.9 ± 3.6 months, while the average time between the infection and T1 was 5.2 ± 3.4 months. Thus, the TTI was 0.82 ± 0.21. We observed a significant increase in FTMTRS total scores from T0 to T1 (FTMTRS total score at T0: 18.9 ± 12.0, T1: 26.9 ± 14.0, *p* = 0.006). Specifically, FTMTRS section B (FTMTRS-B at T0: 8.1 ± 5.8, T1: 11.8 ± 6.8, *p* = 0.004) and section C subscores (FTMTRS-C at T0: 4.3 ± 3.9, T1: 6.7 ± 5.4, *p* = 0.03) significantly increased over time. Conversely, no significant differences were observed for FTMTRS section A subscores between T0 and T1 (FTMTRS-A at T0: 6.5 ± 3.3, T1: 8.4 ± 4.6, *p* = 0.13) (Fig. 2). Overall, we found that 5 out of 13 infected patients deteriorated by more than one SD from the mean baseline FTMTRS total score. Regarding tremor distribution, at T0 all patients had postural and/or kinetic tremor in the upper limbs. Eight of 13 patients (61.54%) had upper limb tremor only, whereas 5 of 13 patients (38.46%) had upper limb tremor in combination with tremor in other body segments. Specifically, 1 of 13 patients (7.69%) had head tremor, 2 of 13 (15.38%) exhibited face tremor, 4 of 13 (30.77%) had voice tremor, while no one exhibited lower limb tremor. At T1, the number of patients with upper limb tremor only decreased to 3 (23.08%), whereas the number of patients with upper limb tremor in combination with tremor in other body segments increased to 10 of 13 patients (76.92%) (*p* = 0.06 by McNemar’s test), showing a trend of tremor spreading. Notably, the number of patients with voice tremor significantly increased to 10 out of 13 (76.92%) at T1 (*p* = 0.03) (Fig. 3). Finally, comparing T0 and T1, the number of body segments affected by tremor increased in infected patients (mean number of body segment affected at T0: 1.5 ± 0.8; T1: 2.9 ± 1.4, *p* = 0.007).

MMSE, HAM-A and HAM-D scores did not change between T0 and T1 (*p* = 0.57, 0.40 and 0.75 respectively).

We found no differences in terms of postural tremor amplitude and frequency as kinematically recorded between T0 and T1 in infected patients (GRMS2 at T0: 0.18, T1: 0.15, *p* = 0.26; Hz at T0: 5.57, T1: 5.42, *p* = 0.33) (Fig. 2). No differences were observed in terms of kinetic tremor amplitude as kinematically evaluated (CI at T0: 1.06, T1: 1.06, *p* = 0.93) (Fig. 2).

**Fig. 4:**
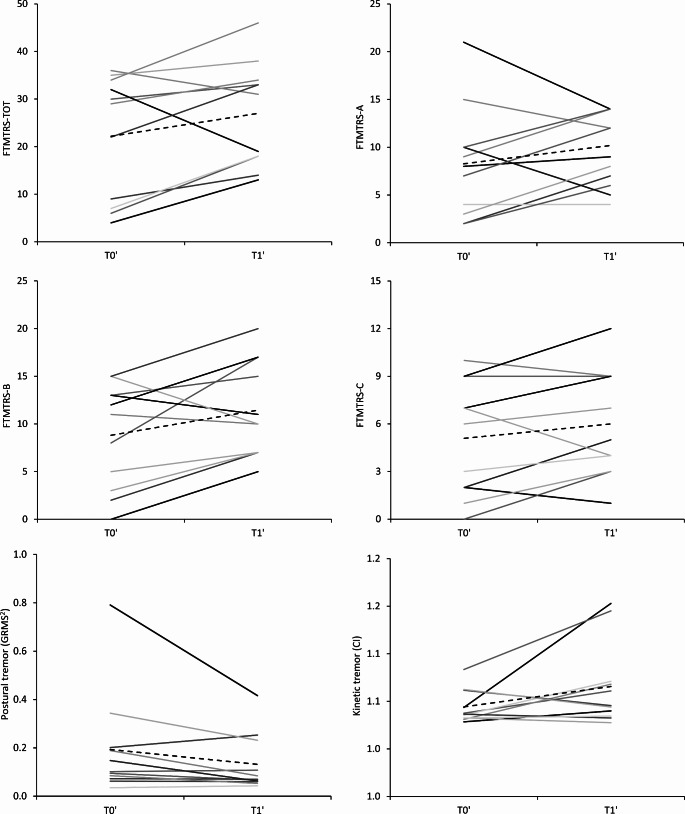
Upper panel: Fahn-Tolosa-Marin Clinical Rating Scale for Tremor (FTMTRS) scores and subscores. Lower panel: postural (left) and kinetic (right) tremor amplitude. Data refers to non-infected patients. Solid lines correspond to individual data and dashed lines to the average value

Among the 11 non-infected ET patients whose data we retrospectively analysed, the average time between T0’ and T1’ was 40.2 ± 8.7 months, thus it was comparable to that observed in the infected subgroup (*p* = 0.57 by Mann–Whitney U test). As opposed to infected patients, however, we did not observe any significant changes in FTMTRS total scores and subscores from T0’ to T1’ in patients who did not contract the infection (FTMTRS total score at T0’: 22.2 ± 13.0, T1’: 27.0 ± 11.0, *p* = 0.11; FTMTRS-A at T0’: 8.3 ± 5.8, T1’: 10.2 ± 4.0, *p* = 0.38; FTMTRS-B at T0’: 8.8 ± 5.5, T1’: 11.5 ± 5.0, *p* = 0.05; FTMTRS-C at T0’: 5.1 ± 3.6, T1’: 6.0 ± 3.4, *p* = 0.15) (Fig. 4). None of the 11 non-infected patients deteriorated by more than one SD from the mean baseline FTMTRS total score (*p* = 0.03 by Fisher’s exact test compared to infected patients). By definition, all patients (100%) at T0’had postural and/or kinetic tremor in the upper limbs. Four of 11 patients (36.36%) had upper limb tremor only, whereas 7 of 11 patients (63.63%) had upper limb tremor in combination with tremor in other body segments. Specifically, 5 of 11 patients (45.45%) exhibited head tremor, 4 of 11 (36.36%) had voice tremor, 1 of 11 (9.09%) had lower limb tremor, whereas no one exhibited face tremor (Fig. 3). At T1’, 1 of 11 patients (9.09%) had upper limb tremor only, whereas 10 of 11 patients (90.91%) had upper tremor in combination with tremor in other body segments (*p* = 0.25 by McNemar’s test as compared to T0’). At T1’, the percentages of patients affected by tremor in specific body parts other than the upper limbs remained stable compared to baseline (*p* values ranging from 0.06 to 0.5). However, the number of body segments affected by tremor in non-infected patients increased between T0’ and T1’ (mean number of body segment affected at T0’: 1.9 ± 0.8; T1’: 3.2 ± 1.4, *p* = 0.006).

We did not find any variation in MMSE (*p* = 0.59), HAM-A nor HAM-D scores between T0’ and T1’ in non-infected patients (*p* = 0.19 and 0.10 respectively).

Consistently with clinical results, we did not find any difference in terms of postural tremor amplitude (*p* = 0.11) and frequency (*p* = 0.93) between T0’ and T1’ (Fig. 4). Again, no differences were observed in terms of kinetic tremor amplitude (CI at T0’: 1.04, T1’: 1.07, *p* = 0.11) (Fig. 4).

Correlation analysis did not reveal any significant correlation between the longitudinal variation of clinical and kinematic data and the TTI in infected patients (r values between 0.11 and 0.47, *p* values between 0.10 and 0.72).

## Discussion

In this study, we explored the potential impact of SARS-CoV-2 infection on tremor in a representative sample of patients with ET. We observed that almost half of the patients who had experienced a SARS-CoV-2 infection reported worsening tremor features. Notably, all patients reporting subjective worsening of tremors also exhibited long COVID symptoms, whereas the prevalence of these symptoms was lower in those without subjective tremor exacerbation. The retrospective clinical data analysis revealed a significant deterioration of tremor in infected patients, expressed as an overall increase of clinical scores due to a greater impact of tremor on motor tasks and to an increased functional disability.

The diagnosis of ET was based on clinical criteria [18]. However, the enrolled patients have been consistently followed in the outpatient clinic for an extended period, instilling confidence in the clinical diagnosis of ET. Clinical and kinematic evaluations were conducted after discontinuing tremor medications, eliminating the potential influence of medication on our results. While acknowledging the inherent variability of tremor, influenced by emotional or stressful factors, we sought to minimize variability by conducting the evaluations in a consistent laboratory setting and at the same time of day. [15,16] In addition, cognitive and psychiatric evaluations on patients did not reveal any changes, thus ruling out the involvement of these symptoms in influencing the tremor. Moreover, the researchers involved in the collection of clinical and kinematic data were not involved in data analysis and processing. Finally, the retrospective analysis of clinical and kinematic data was executed by researchers who were blinded to the occurrence of SARS-CoV-2 infection.

The first notable finding in this study is that many patients with ET reported a subjective worsening of tremor following SARS-CoV-2 infection. The worsening consisted in most of the cases of an increase in tremor amplitude and duration throughout the day, and, in one case, the self-reported spread of tremor in a previously unaffected body part. Retrospective analysis of clinical data confirmed that tremor severity increased in infected patients, with tremor spread in different body segments and a worsening of tremor during specific complex tasks (i.e., drawing spirals and several activities of daily living). In contrast, FTMTRS section A did not show significant differences between the two evaluations. It is possible that its dependence on the amplitude of tremor under different activation conditions, as well as its distribution, prevented statistical significance from being achieved. Furthermore, the limited sample size should be taken into account in the analysis of these results. Interestingly, this overall deterioration was not observed in non-infected patients, whose clinical scores did not significantly increase between the two assessments, although a topographical spread of tremor was demonstrated also in this subgroup, confirming previous observation indicating a progression of the pathology over the years [26, 29–31]. This finding therefore suggests a worsening of ET following infection, which goes beyond the progression of tremor over time that is usually observed in non-infected cases. The exacerbation of motor symptoms after SARS-CoV-2 infection in patients with other movement disorders had been documented in previous studies [12, 32–35]. Several mechanisms have been proposed to explain this finding, including a direct viral effect on the nervous system or post-infectious immune-mediated diseases of the brain [33, 36–39]. In ET, only two cases of tremor worsening after COVID-19 have been previously described and it was hypothesized, that SARS-CoV-2 infection initiates an immune-mediated inflammatory response in the central nervous system [40–42]. This, in turn, leads to a dysfunction within the cerebellar circuitry, potentially exacerbating tremors in individuals with ET [15, 16]. The clinical data from the present study likely support this hypothesis.

The tremor worsening reported by the patients and observed at clinical assessment was mainly related to a tremor increase during complex actions, e.g. drawing, pouring, and its impact on the daily living activities (as evaluated by the sections B and C subscores of the FTMRS). In contrast, clinical and kinematic data showed substantial stability in tremor characteristics, including amplitude and frequency, in both infected and non-infected ET patients. Although there was some heterogeneity in the data, with individuals among the infected patients presenting a clear kinematic worsening, this was not statistically significant at the group level. One could therefore speculate that SARS-CoV-2 infection may have a more pronounced detrimental effect on complex motor functions that require finer cerebellar control [43], though this is speculative and requires further investigation.

Another study result consisted of the substantial percentage of ET patients reporting long COVID-19 symptoms. Long COVID is a prevalent complication of SARS-CoV-2 infection. The majority of cases occur in non-hospitalized patients with mild COVID-19 symptoms [9]. Although the evidence is still limited, various pathophysiological mechanisms are believed to be associated with long COVID [44], including immune dysregulation, microbiota disruption, and persisting reservoirs of SARS-CoV-2 in tissues [9, 44–47]. Notably, all ET patients who reported a subjective worsening of tremor also experienced long COVID symptoms. Nonetheless, while these symptoms encompass anxiety, depression, and cognitive disorders, many other reported long COVID symptoms are not adequately assessed by the clinical scales employed (HAM-A, HAM-D, and MoCA), which indeed did not show any significant variations between the two evaluations. This finding aligns with previous studies demonstrating a worsening of motor function only in patients with PD who developed long COVID [45]. We can hypothesize that long COVID contributes to a greater perception of the impact that tremor has on activities of daily living in patients, without, however, being reflected in an objective worsening of the tremor as assessed by kinematic analysis.

The present study has some limitations that need acknowledgment. Firstly, the sample size is relatively limited. Nevertheless, this study represents the most extensive case study examining the effects of SARS-CoV-2 infection on ET. Also, the retrospective design of the study introduces potential biases, particularly recall bias. However, we believe that the retrospective study was the most suitable approach for this study, effectively minimizing potential bias due to improper blinding. Additionally, the use of the consensus criteria for the diagnosis of ET has limitations in terms of specificity, however they are the most widely applied and most reliable criteria to date. The variability in the time elapsed between the infection and assessments must be also considered when interpreting our results. Additionally, the potential presence of asymptomatic or undiagnosed SARS infections introduces the possibility that some individuals categorized as unaffected may have been exposed to the virus without displaying symptoms or being diagnosed. Again, none of the enrolled patients reported severe symptoms due to SARS-CoV-2 infection, emphasizing the need for further studies to determine whether severe COVID-19 may have different effects on tremor in ET. Note that the data refer to virus variants circulating between 2018 and 2023 [48] and do not necessarily reflect those circulating at current times.

In conclusion, this study contributes significantly to understanding the potential long-term neurological implications of SARS-CoV-2 infection in ET patients. We here provide a comprehensive analysis of the impact of SARS-CoV-2 infection in patients with ET, highlighting a potential correlation between viral infection and worsening of tremor. We also found that long COVID may be one of the major contributors to tremor deterioration, through an increased impact on common activities of daily living in patients. Further research with larger samples and integrated approaches, combining clinical and kinematic data, is crucial to confirm and deepen these observations.

## Data Availability

The data that support the findings of this study are available on request from the corresponding author.

## Abbreviations

CI: Curvature Index
COVID-19: Coronavirus Disease 2019
ET: Essential Tremor
FTMTRS: Fahn-Tolosa-Marin Clinical Rating Scale for Tremor
GRMS^2^: Root Mean Square of the Acceleration Traces
HAM-A: Hamilton Anxiety Rating Scale
HAM-D: Hamilton Depression Rating Scale
MMSE: Mini-Mental State Examination
P1: Posture 1
P2: Posture 2
PD: Parkinson’s disease
SARS-CoV-2: Severe Acute Respiratory Syndrome Coronavirus 2
SD: Standard Deviation
TTI: Time to infection
